# Mechanical Strength and Inhibition of the *Staphylococcus aureus* Collagen-Binding Protein Cna

**DOI:** 10.1128/mBio.01529-16

**Published:** 2016-10-25

**Authors:** Philippe Herman-Bausier, Claire Valotteau, Giampiero Pietrocola, Simonetta Rindi, David Alsteens, Timothy J. Foster, Pietro Speziale, Yves F. Dufrêne

**Affiliations:** a Institute of Life Sciences, Université catholique de Louvain, Louvain-la-Neuve, Belgium; b Department of Molecular Medicine, Unit of Biochemistry, University of Pavia, Pavia, Italy; c Department of Microbiology, Trinity College Dublin, Dublin, Ireland; d Walloon Excellence in Life Sciences and Biotechnology (WELBIO), Wallonia, Belgium

## Abstract

The bacterial pathogen *Staphylococcus aureus* expresses a variety of cell surface adhesion proteins that bind to host extracellular matrix proteins. Among these, the collagen (Cn)-binding protein Cna plays important roles in bacterium-host adherence and in immune evasion. While it is well established that the A region of Cna mediates ligand binding, whether the repetitive B region has a dedicated function is not known. Here, we report the direct measurement of the mechanical strength of Cna-Cn bonds on living bacteria, and we quantify the antiadhesion activity of monoclonal antibodies (MAbs) targeting this interaction. We demonstrate that the strength of Cna-Cn bonds *in vivo* is very strong (~1.2 nN), consistent with the high-affinity “collagen hug” mechanism. The B region is required for strong ligand binding and has been found to function as a spring capable of sustaining high forces. This previously undescribed mechanical response of the B region is of biological significance as it provides a means to project the A region away from the bacterial surface and to maintain bacterial adhesion under conditions of high forces. We further quantified the antiadhesion activity of MAbs raised against the A region of Cna directly on living bacteria without the need for labeling or purification. Some MAbs are more efficient in blocking single-cell adhesion, suggesting that they act as competitive inhibitors that bind Cna residues directly involved in ligand binding. This report highlights the role of protein mechanics in activating the function of staphylococcal adhesion proteins and emphasizes the potential of antibodies to prevent staphylococcal adhesion and biofilm formation.

## IMPORTANCE

Cna is a collagen (Cn)-binding protein from *Staphylococcus aureus* that is involved in pathogenesis. Currently, we know little about the functional role of the repetitive B region of the protein. Here, we unravel the mechanical strength of Cna in living bacteria. We show that single Cna-Cn bonds are very strong, reflecting high-affinity binding by the collagen hug mechanism. We discovered that the B region behaves as a nanospring capable of sustaining high forces. This unanticipated mechanical response, not previously described for any staphylococcal adhesin, favors a model in which the B region has a mechanical function that is essential for strong ligand binding. Finally, we assess the antiadhesion activity of monoclonal antibodies against Cna, suggesting that they could be used to inhibit *S. aureus* adhesion.

## INTRODUCTION

*Staphylococcus aureus* expresses a variety of cell surface proteins, including the microbial surface components recognizing adhesive matrix molecules (MSCRAMMs), that bind to host extracellular matrix proteins (1). The collagen (Cn)-binding protein Cna is a prototype of the MSCRAMMs that has an important role in staphylococcal pathogenesis, both as an adherence factor and as an immune evasion factor. Cna is a proven virulence factor in septic arthritis, where the strength of adhesion to collagen correlates with disease pathogenesis (1, 2). In addition, Cna binds to complement protein C1q and prevents the classical pathway of complement fixation (3). It is therefore of great biological significance to understand the molecular basis of the Cna-Cn interaction.

**FIG 1:**
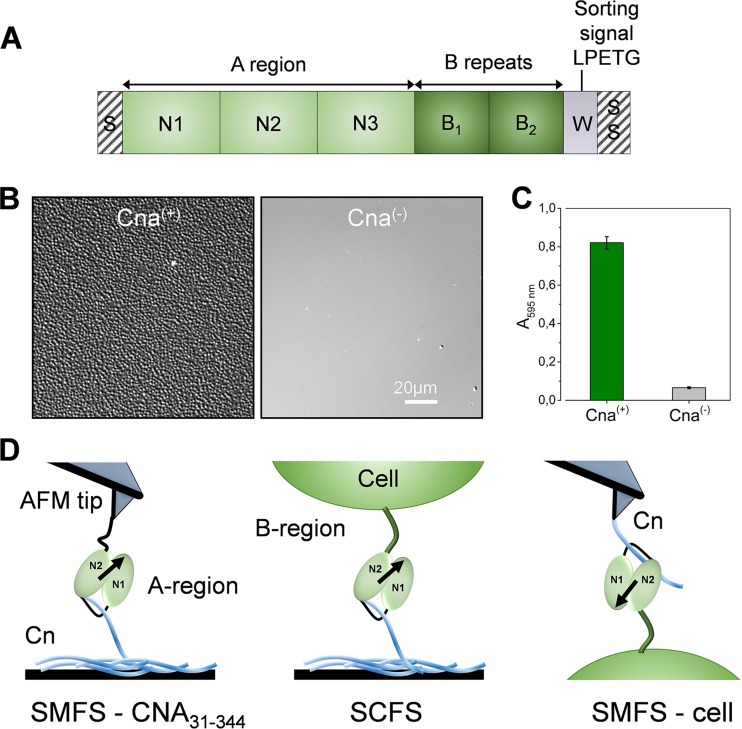
Studying Cna-mediated adhesion. (A) Schematic structure of Cna showing the ligand-binding A region made of the N1N2N3 subdomains (beginning at residues 31, 140, and 344, respectively) and the repeated B region consisting of two B-repeat units (beginning at residues 535 and 722) and whose functional role is unclear. The number of B repeats is strain dependent: the Phillips strain used here has 2 B repeats, whereas the one used in structural studies has 3 repeats (5). (B) Microscale adherence assay using optical microscopy. Images of *S. aureus* Phillips bacteria expressing or not expressing full-length Cna [Cna^(+)^ and Cna^(−)^ cells] following adhesion to type II collagen (Cn)-coated substrates are shown. (C) Macroscale adherence assay using crystal violet. Microtiter wells coated with Cn were incubated with bacteria, rinsed, and stained with crystal violet, and the absorbance at 595 nm was measured in an ELISA plate reader. Means and SD of results of two independent experiments, each performed in triplicate, are presented. (D) Atomic force microscopy analysis of the Cna-Cn interaction *in vitro* and *in vivo*. (Left) Single-molecule force spectroscopy (SMFS) of recombinant CNA_31–344_, i.e., a portion of the A region which contains the ligand-binding domains. (Middle and right) Single-cell force spectroscopy (SCFS; middle) and SMFS (right) of full-length Cna expressed in *S. aureus* Phillips bacteria (for the sake of clarity, the N3 domain is not shown in the middle and right cartoons). As illustrated, Cna binds to Cn via the collagen hug mechanism, where the N1N2 subdomains cooperate to wrap around the rope-like structure of collagen.

Cna binds to its ligand using a variation of the high-affinity dock lock and latch (DLL) mechanism (4), known as the “collagen hug” (5). This multistep binding mechanism involves two subdomains that cooperate to wrap around and “hug” the rope-like structure of a collagen monomer. The ligand-binding domains are in the N-terminal A region of Cna (6), made of three subdomain structures, N1, N2, and N3 (Fig. 1A). The N1 and N2 subdomains are variants of the IgG fold which are predominantly composed of two antiparallel β-sheets. The long linking peptide that connects N1 and N2 forms a hole at the interface between the two domains in which a collagen triple-helical rod can be accommodated. The ligand docks into a shallow trench in the N2 subdomain. This is followed by a conformational change that enables the linker between N1 and N2 to wrap around the collagen molecule. Finally, the C-terminal latch of N2 binds to N1 to secure the ligand in place. Despite the biological importance of the collagen hug mechanism, the molecular forces involved have never been measured.

Following the A region is the repetitive B region made of multiple repeat units (Fig. 1A), but whether it plays a functional role remains an unsolved problem. B repeats have been suggested to play roles in the stability and function of the A region and in projecting the A region away from the cell surface (7, 8). Structural analysis showed that the B region has a novel fold, the CnaB fold, that is reminiscent of but is in an inverse orientation with respect to the IgG fold (9). It was proposed that the B domain of Cna could function as a stalk that would help in presenting the A region away from the bacterial cell surface. Modeling of the B repeats showed that these domains pack in a zig-zag fashion, suggesting that they might stretch and retract from the bacterial cell wall (9). While the notion of such a role is appealing, it has never been demonstrated.

Antiadhesion therapy is a promising alternative to antibiotics for fighting biofilm infections caused by multiresistant bacteria (10, 11). Staphylococcal surface proteins have been shown to be efficient targets for antibody-mediated strategies against adhesion and biofilms (12, 13). A series of monoclonal antibodies (MAbs) raised against Cna interfered with the attachment of bacteria to collagen substrates, suggesting that they could be used as therapeutic agents (14). The design of antiadhesion compounds would clearly benefit from novel assays for the fast, label-free screening of the most efficient molecules and for understanding their mechanisms of action.

In recent years, atomic force microscopy (AFM) has been increasingly used to study the molecular basis of staphylococcal adhesion (15–22), providing novel insight into the binding mechanisms of MSCRAMMs. So far, however, the technique has never been applied to investigate the binding strength and molecular elasticity of Cna. Here, we used single-cell and single-molecule AFM to measure the mechanical strength of Cna in living bacteria and to determine the contribution of the B region to the protein mechanics. We show that single Cna-Cn bonds are much stronger than those generally measured for bacterial adhesins, consistent with the high-affinity collagen hug mechanism. The B region is required for strong binding and displays nanospring properties that, we believe, fulfill an important function: by acting as a rigid spring, the B repeats aid Cna to project away from the cell surface and to maintain bacterial adhesion even under conditions of high mechanical stress. We also quantify the antiadhesion activity of a series of MAbs against the ligand-binding A region, showing that some are more efficient than others in blocking *S. aureus* adhesion.

## RESULTS

### Bacterial adhesion to collagen-coated substrates.

To study the Cna-binding forces, we used cells of *S. aureus* strain Phillips [referred to here as Cna^(+)^ cells] and its isogenic mutant [Cna^(−)^ cells] (2). We confirmed the expression of functional adhesins using microscale and macroscale adhesion assays. Both optical microscopy imaging (Fig. 1B) and crystal violet staining (Fig. 1C) showed that Cna^(+)^ cells adhered in large amounts to type II Cn-coated substrates, while no (or little) adhesion was observed with Cna^(−)^ cells. These data confirm that the protein is well exposed at the cell surface and shows strong Cn-binding activity.

### Recombinant fragments of the ligand-binding region show moderate binding strength.

**FIG 2:**
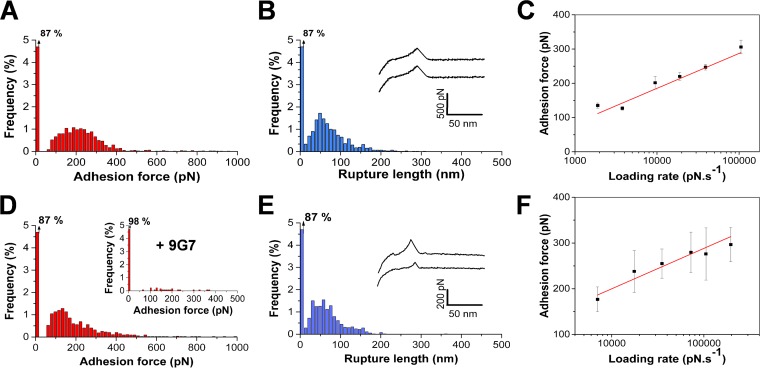
Single-molecule force spectroscopy shows that recombinant CNA_31–344_ binds collagen with moderate strength. (A and B) Adhesion force histogram (B) and rupture length histogram (B), with representative retraction force profiles obtained by recording force-distance curves in PBS between CNA_31–344_ tips and Cn substrates. CNA_31–344_ fragments were immobilized on the tips using a PEG-benzaldehyde linker. Data were pooled from results of independent experiments performed using 8 different tips and substrates. (C) Dependence of the adhesion force on the loading rate applied during retraction, measured between CNA_31–344_ tips and Cn substrates (means ± standard errors of the means [SEM]). Similar plots were obtained in independent experiments. (D to F) Force data obtained using the inverse configuration, in which Cn molecules were immobilized to the tips using the NHS surface chemistry. Data in panels D and E were pooled from results of independent experiments performed using 6 different tips and substrates. The inset in D shows the data obtained after addition of 9G7 monoclonal antibodies (10 µg ml^−1^). That the errors in panel F are larger than those in panel C simply reflects the notion that fewer data points were recorded. Loading-rate plots similar to those shown in panel F were obtained in independent experiments. All curves were obtained using a contact time of 250 ms, a maximum applied force of 250 pN, and approach and retraction speeds of 1,000 nm s^−1^, except in panels C and F, for which the retraction speed was varied.

We initially investigated the binding strength of the ligand-binding A region of Cna in the absence of other staphylococcal components, using single-molecule force spectroscopy (SMFS; Fig. 1D). Force-distance curves were recorded between substrates coated with type II Cn and AFM tips functionalized with recombinant fragments corresponding to the predicted N1N2 subdomains (amino acid residues 31 to 344; here, CNA_31–344_). Cn was attached via *N*-hydroxysuccinimide (NHS) surface chemistry, whereas purified CNA_31–344_ protein constructs were immobilized at low density to the tip using a polyethylene glycol (PEG)-benzaldehyde linker known to favor single-molecule detection (23). In Fig. 2A and B, we present the average force data that we obtained for a total of 8 independent tips and substrates, i.e., the adhesion force and rupture length histograms, together with representative retraction force curves. A substantial fraction (13%) of the force profiles featured well-defined adhesion peaks with a mean adhesion force of 218 ± 86 pN and a rupture length of 77 ± 45 nm (means ± standard deviations [SD], from a total of 7,909 recorded force curves). As a control, we tested the inverse configuration, i.e., by attaching Cn to the tip and Cna to the substrate using NHS surface chemistry (Fig. 2D and E). There were larger variations in the data, presumably reflecting differences in surface chemistry. Nevertheless, similar adhesion forces and rupture lengths were observed (195 ± 108 pN and 71 ± 39 nm; 6 independent tips and substrates). Addition of the 9G7 monoclonal antibody raised against the minimal ligand-binding domain (residues 151 to 318) abolished most adhesion events (Fig. 2D, inset), thus providing evidence that specific CNA_31–344_-Cn interactions were probed. We also analyzed CNA_31–531_ fragments corresponding to the full-length A region (see Fig. S1 in the supplemental material), thus containing the N1N2N3 subdomains, and we found adhesion forces that were in the same range (195 ± 149 pN) and yet had substantially more variability and lower adhesion frequency (4%). These poorer binding properties may be related to earlier surface plasmon resonance (SPR) measurements showing that the CNA_31–531_ protein construct binds Cn with lower affinity than CNA_31–344_ (5). A possible explanation is that the shorter CNA_31–344_ fragment may have easier access to its ligand.

Intriguingly, the ~220-pN force of CNA_31–344_ is much lower than the ~2 nN force measured for single bonds between the *S. epidermidis* SdrG adhesin and the blood plasma protein fibrinogen (Fg) (22), which involves the DLL mechanism in which dynamic conformational changes in the A region lead to greatly stabilized complexes (4). As the collagen hug shows strong similarities with the DLL mechanism, this suggests that CNA_31–344_ alone is not sufficient to give rise to strong interactions. At first sight, this is in contrast with SPR and enzyme-linked immunosorbent assay (ELISA) data showing that CNA_31–344_ binds collagen with high (~0.2 µM) affinity (5). The origin of this discrepancy is unclear but could be related to the fact that the earlier analyses used soluble protein fragments whereas here CNA_31–344_ was immobilized to the tip. It is possible that when the ligand-binding domain is anchored to a surface without the B region, only the first step of the collagen hug mechanism, i.e., binding of the triple-helical collagen molecule into the trench, is achieved, further conformational changes being hindered due to limited molecular mobility. We also note that the mean rupture length, ~80 nm, is shorter than the length increment expected for the CNA_31–344_-Cn complex. As Cn proteins were attached to the substrate via multiple sites (NHS chemistry), we expected that it would not substantially contribute to the measured extensions. Considering that each amino acid residue contributes 0.36 nm to the contour length of a fully extended polypeptide chain and that the folded length of CNA_31–344_ is ~8 nm (7), the increase in length for an unfolded CNA_31–344_ should be ~105 nm. This means that the N1N2 subdomains are not fully unfolded when the complex is stretched.

We assessed the dissociation rate of the bond by measuring the dependence of the binding strength on the loading rate (Fig. 2C). The mean adhesion force (*F*) increased linearly with the logarithm of the loading rate (*r*) as observed for other receptor-ligand systems (24). The length scale of the energy barrier, *x_β_*, was assessed from slope *f_β_* of the plot of *F* versus ln(*r*) and found to be *x_β_* = 0.1 nm, in the range of values typically measured by single-molecule AFM. Extrapolation to zero forces yielded the kinetic off-rate constant of dissociation at zero force: *k*_off_ = *r*_F = 0_ *x*_β_/*k*_B_*T* = 2 × 10^−4^ s^−1^. Independent experiments led to values that were in the same range (i.e., that did not differ by more than an order of magnitude). Changing the surface chemistry did not substantially change the loading rate dependence and *k*_off_ values (Fig. 2F [*k*_off_ = 5 × 10^−4^ s^−1^]), supporting the validity of the measurements of these dynamic forces. The off-rate value suggests that the bond associated with the first step of the collagen hug dissociates rather slowly, which does not contradict SPR and ELISA data (5).

### Cna on living bacteria mediates weak and strong interactions.

**FIG 3:**
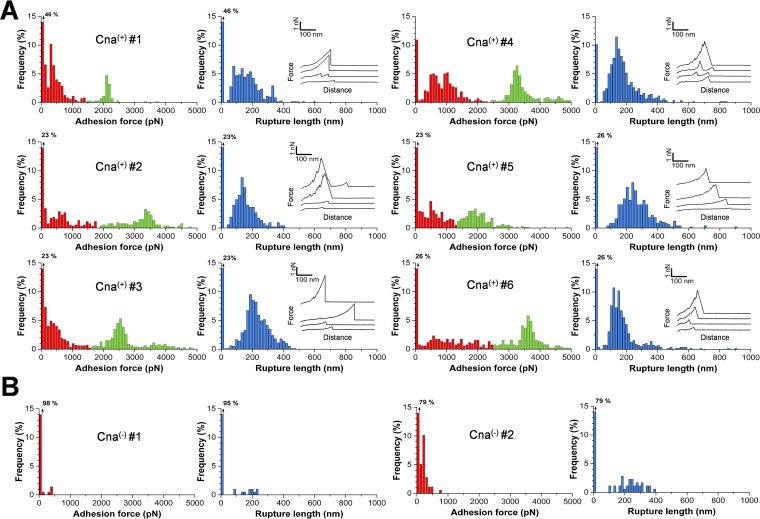
Single-cell force spectroscopy captures the strength of Cna-Cn bonds in living bacteria. (A) Adhesion force (left) and rupture length (right) histograms with representative retraction force profiles obtained by recording force-distance curves in PBS between six different Cna^(+)^ cells and Cn substrates. (B) Force data obtained under the same conditions for two Cna^(−)^ cells. All curves were obtained using a contact time of 100 ms, a maximum applied force of 250 pN, and approach and retraction speeds of 1,000 nm s^−1^.

We then probed the mechanical strength of Cna-Cn bonds using full-length adhesins expressed on living *S. aureus* cells. A bacterium was attached to an AFM cantilever, and force-distance curves were recorded between the bacterial probe and a Cn substrate using single-cell force spectroscopy (SCFS; Fig. 1D) (25, 26). Figure 3A shows the adhesion forces and rupture lengths measured for six representative Cna^(+)^ cells (including cells from independent cultures). Most (54% to 89%) of the force curves featured well-defined adhesion force peaks ranging from ~100 to 5,000 pN, with a rupture length of 189 ± 100 nm (means ± SD; *n* = 3,019 adhesive curves). These forces were essentially missing on Cna^(−)^ cells (Fig. 3B), demonstrating that they were due to specific Cna-Cn interactions.

**FIG 4:**
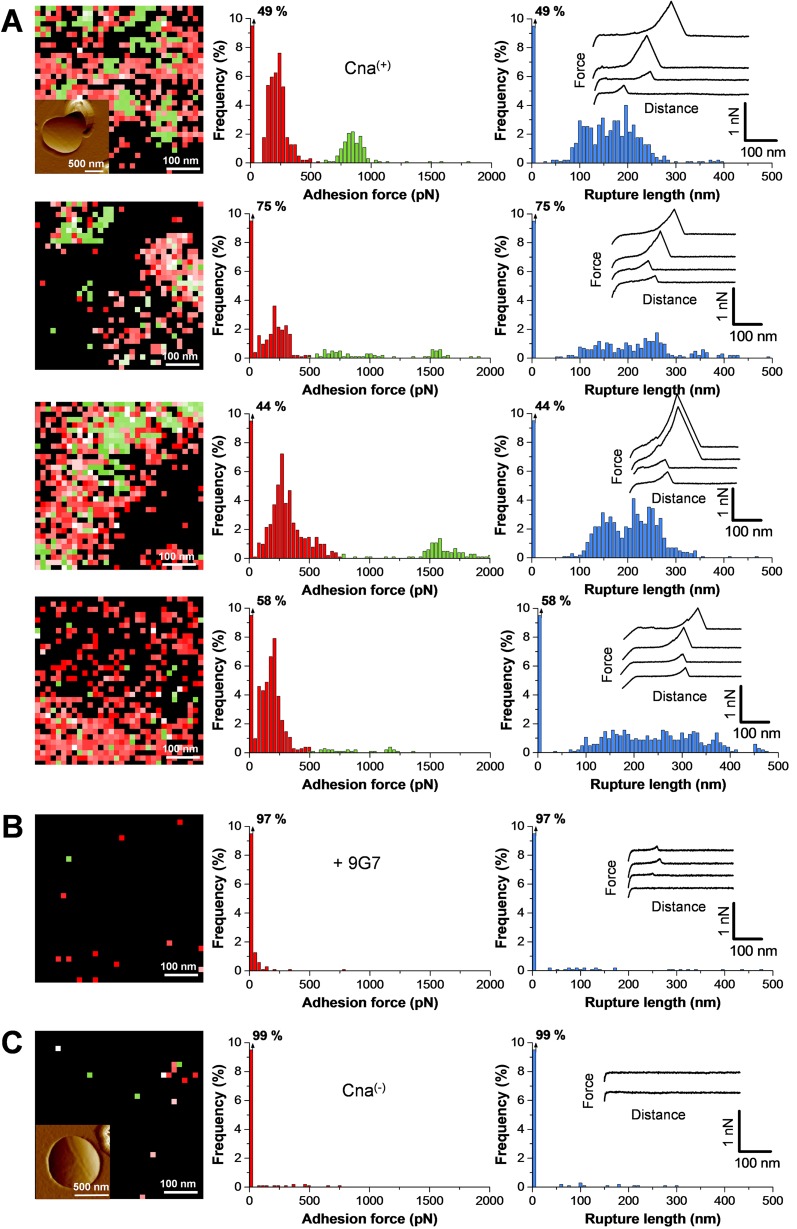
Localization and binding strength of single Cna proteins on the bacterial cell surface. (A) Adhesion force maps (color scale, 2,000 pN) and adhesion force profiles and rupture length histograms with representative retraction force profiles obtained by recording force curves in PBS across the surface of four Cna^(+)^ cells using tips labeled with Cn. The inset in the upper map is a deflection image of the cell surface. The red and green colors highlight the detection of weak (<500 pN) and strong (>500 pN) binding events. (B) Force data obtained on a Cna^(+)^ cell following injection of 9G7 monoclonal antibodies (10 µg ml^−1^). (C) Force data obtained on a Cna^(−)^ cell in PBS. For panels B and C, duplicate experiments led to similar results. All curves were obtained using a contact time of 100 ms, a maximum applied force of 250 pN, and approach and retraction speeds of 1,000 nm s^−1^.

Close inspection of the force histograms (Fig. 3A) revealed two types of adhesive interactions, i.e., weak forces of 670 ± 368 pN (*n* = 1,478) and strong forces of 3,155 ± 1,217 pN (*n* = 1,543). Although the strong forces differed from one cell to another, their distribution data were sharply defined and centered at 2,124 ± 140 pN, 3,377 ± 197 pN, 2,504 ± 170 pN, 3,254 ± 145 pN, 1,893 ± 326 pN, and 3,617 ± 175 pN for cell no. 1 to cell no. 6, respectively. This leads us to believe that, for a given cell, the same numbers of Cna-Cn complexes were probed from one curve to another. The high forces were in the range of those measured for DLL-based SdrG-Fg interactions using the same SCFS assay (22). Thus, the strong forces observed here are consistent with a high-affinity interaction, such as the collagen hug. As cells displayed high force levels ranging from ~2 to 4 nN, we hypothesize that they involve multiple high-affinity bonds of ~1-nN unit force, as supported by our single-molecule analyses (Fig. 4).

Rupture lengths were in the same range for both weak and strong bonds (~190 nm) and showed only small variations in comparisons of different cells, thus supporting the idea that the same bonds were reproducibly probed. The rather short extensions were much smaller than those measured for fully extended Cna molecules. Considering that the Cna molecule is made of 1,000 residues and has a folded length of ~20 nm (7), full unfolding of the protein should give an extension of ~340 nm, longer than that observed here. In our reasoning, we consider that the contribution of the linker and bacterial cell wall to the rupture length can be neglected. Interestingly, the ~190-nm extensions agree reasonably well with the expected 500 residues of the A domain. These observations led us to postulate that perhaps the B region is too rigid to be unfolded, even in the presence of high forces, because of its specific structural properties. Cna B domains present in various surface proteins from Gram-positive bacteria feature intramolecular isopeptide bonds that stabilize the mechanical properties of secreted proteins (27, 28). These covalent cross-links are formed autocatalytically and are thought to play a role in helping piliated bacteria to resist shear stresses. Confirming this view, pulling experiments performed on the Spy0128 major pilin from *Streptococcus pyogenes* showed that the protein is inextensible owing to isopeptide bonds (29). As there is evidence that CnaB domains from *S. aureus* adhesins could contain isopeptide bonds (30), we speculate that they may play a role in the mechanical stabilization of the Cna structure.

### Single adhesins form cell surface nanodomains and show strong binding forces.

To understand the localization and mechanical strength of individual Cna molecules, living bacteria were probed by SMFS with Cn-modified tips (Fig. 1D). Shown in Fig. 4A are the adhesion force maps as well as the distributions of the adhesion forces and rupture lengths obtained between Cn tips and four Cna^(+)^ cells, including cells from independent cultures. As can be seen, the curves displayed dual force distributions with single adhesion peaks and either weak forces, of 239 ± 107 pN, or strong forces, of 1,166 ± 544 pN (*n* = 2,350 adhesive curves). A major drop in the probability of adhesion was observed in blocking the cells with free 9G7 MAbs (Fig. 4B) or using Cna^(−)^ mutant cells (Fig. 4C), thus demonstrating the specificity of the probed interactions. The weak forces (~240 pN) were in the range of those measured with purified CNA_31–344_ (~220 pN; Fig. 2A), suggesting that they represent the first step of the collagen hug, that is, hydrophobic interactions between collagen and residues in the shallow trench of the N2 domain (5). As with whole cells (Fig. 3), full-length Cna featured strong forces never observed on CNA_31–344_ (Fig. 2) or CNA_31–531_ (see Fig. S1 in the supplemental material), suggesting that the B domain plays a role in enabling strong bonds to form. We attribute the ~1,200-pN force to the rupture of single complexes fully stabilized by the collagen hug. That single bonds were probed is supported by several observations. First, strong forces were never observed using CNA_31–344_ (Fig. 2) or CNA_31–531_ (see Fig. S1) fragments. Second, these forces showed a rather narrow force distribution and featured single rupture peaks. If multiple bonds were probed, one would expect to observe a wider range of forces corresponding to multiples of the weak unit force and/or multiple rupture peaks, which was not the case. The rupture lengths (200 ± 30 nm) were close to the values obtained on whole cells by SCFS (Fig. 3), suggesting again that only the A region is unfolded.

Adhesion maps revealed that Cna was largely exposed on the cell surface, with some variations in protein density from one cell to another. Adhesins were assembled into nanometer-scale domains (see red pixels), a behavior reminiscent of the SdrG cell surface distribution (22). Interestingly, within these domains, strong binding events resulted in localized patches (green pixels) that we attribute to the clustering of single Cna-Cn complexes engaged in the collagen hug, possibly in a cooperative manner. As the clustering of Cna may play a role in strengthening adhesion, these results emphasize the need to probe individual Cna molecules at high spatial resolution and in their fully functional cellular context. In contrast to *in vitro* assays, such as isothermal titration calorimetry and surface plasmon resonance, AFM is the only technique that can localize and functionally analyze adhesins directly on the surface of live cells.

### The B region has a mechanical function that is required for strong ligand binding *in vivo*.

**FIG 5:**
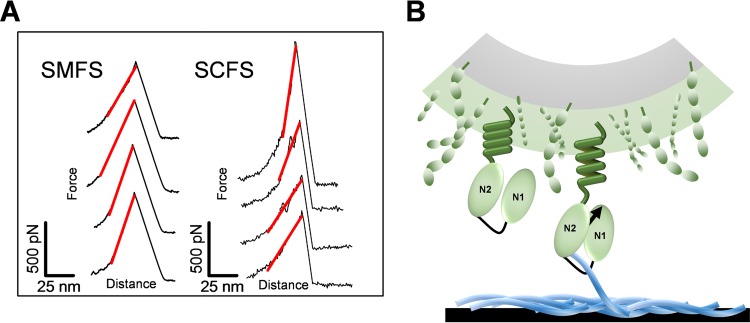
Nanospring properties of Cna. (A) Representative stretching curves obtained by SMFS (left) and SCFS (right), documenting well-defined adhesion peaks with a Hookean spring behavior. (B) The B region has a previously unidentified mechanical function that is essential for activating Cn binding *in vivo*. The spring properties of the B region trigger the projection of the ligand-binding region beyond other surface components, thus enabling the collagen hug and helping to maintain bacterial adhesion under conditions of high mechanical forces.

Another important outcome of our experiments was that Cna featured unusual mechanical properties (Fig. 5). When modular proteins, such as titin and fibronectin, are loaded with mechanical force, their secondary structures (α-helices, β-sheets) unfold, giving rise to nonlinear adhesion force peaks that are well described by the worm-like-chain (WLC) model (31, 32). In contrast, the strong force peaks observed here by SCFS (Fig. 3) and SMFS (Fig. 4) were not fitted with a WLC model. Rather, the forces were proportional to distance, indicating that the stretched proteins behave as linear springs (Fig. 5A). We consider that the bacterial cell wall, which is a very stiff structure, does not contribute significantly to the observed mechanical response. Using the slope (*s*) of the linear portion of the raw deflection versus piezo displacement curves and the equation *k_p_* = (*k*_c_ × *s*)/(1 − *s*), where *k*_c_ is the spring constant of the AFM cantilever and *k*_p_ the spring constant of the protein, we found that, for a single Cna probed by SMFS, *k_p_* = 15 ± 5 pN nm^−1^ (from *n* = 103 force peaks). The variability in protein spring constants may be due to lateral intermolecular interactions between neighboring adhesins, as also evidenced by our single-molecule images documenting protein clustering (Fig. 4). The curves obtained by SCFS yielded constants corresponding to stiffer springs, with *k_p_* = 30 ± 17 pN nm^−1^ (*n* = 50), in line with the notion that when multiple protein springs are loaded in parallel, not only the bond strength (33) but also the spring constant will increase (the equivalent spring constant for two springs in parallel is the sum of their individual spring constants). Indeed, peaks with the strongest forces generally corresponded to the highest spring constants.

We believe that the unusual mechanical response of Cna seen *in vivo* is associated with the B region because linear force peaks were not observed with recombinant CNA_31–344_ (Fig. 2); in addition, the rupture lengths measured for full-length Cna by SCFS (~190 nm) and SMFS (~200 nm) were clearly shorter than that expected for fully unfolded proteins (~340 nm) but matched the unfolding of the 500-residue A domains. So our experiments showed that the B region behaves as a nanospring *in vivo* which can withstand high forces (~1,200 pN) without being unfolded, while forces of 150 to 300 pN are usually sufficient to unfold β-fold domains (32). This mechanical stiffness is in agreement with the unusual structure of the CnaB fold, where internal isopeptide bonds might stabilize the protein mechanical properties (27, 28).

Our finding is reminiscent of the properties of the Wsc1 yeast sensor, which has been shown to behave as a linear spring capable of resisting high mechanical force and of responding to cell surface stress (34). Using single-molecule AFM, the stiff, rod-like structure of the sensor was shown to result from protein glycosylation. The results were in favor of a model where Wsc1 would function as a mechanosensor capable of detecting mechanical forces acting on the cell wall. Similarly, one may ask whether Cna stiffness fulfills a dedicated function. The direct correlation between the occurrence of strong bonds and spring-like properties leads us to believe that this mechanical behavior plays a functional role (Fig. 5B). The ability of the B region to behave as a spring able to withstand large mechanical loads makes it ideally suited to favor the projection, exposure, and clustering of the ligand-binding region. The strong mechanical stability of Cna might aid the protein to maintain its adhesive function even under conditions of high physiological shear stresses.

### Antiadhesion activity of monoclonal antibodies.

Immunization with recombinant fragments of Cna has been shown to protect mice against *S. aureus*-induced septic death (1), suggesting that Cna could be used as a vaccine to prevent staphylococcal infections. We therefore used our SCFS assay to study the ability of a series of MAbs raised against the minimal ligand-binding domain (14) to efficiently block the adhesion of *S. aureus* to Cn substrates. The idea is that binding of MAbs to the ligand binding region may interfere with the Cna-Cn interaction, thus inhibiting bacterial adhesion. Yet, as the different MAbs recognize epitopes that are distributed throughout the adhesin structure (14), their inhibition activity may greatly vary. Thus, there is a need for new assays capable of assessing the efficiency of such antiadhesion compounds.

**FIG 6:**
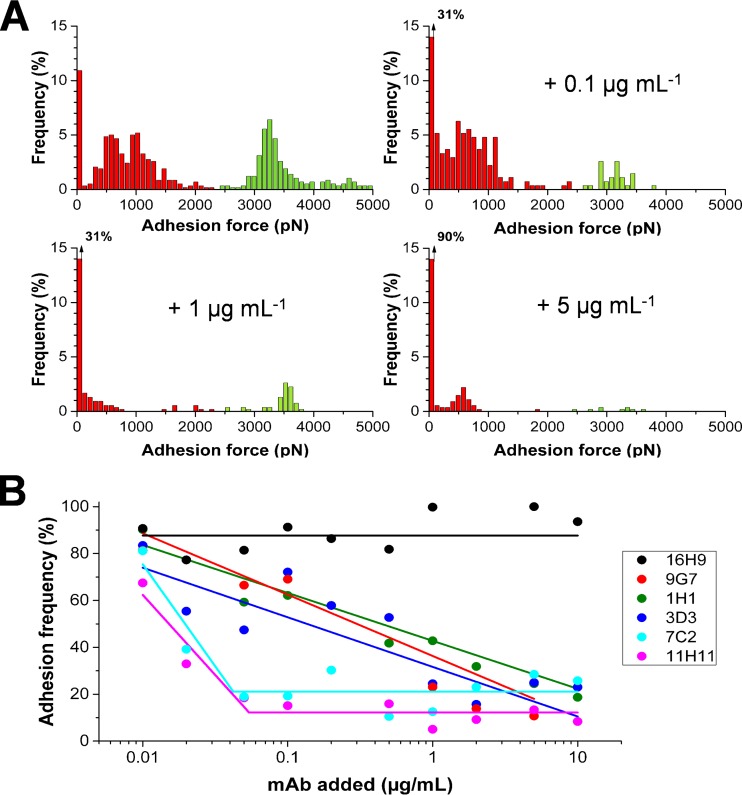
Inhibition of single-cell adhesion by anti-Cna monoclonal antibodies. (A) Variation of the distribution of adhesion forces measured by SCFS between a Cna^(+)^ cell and a Cn substrate upon addition of the 9G7 MAb at increasing concentrations. Similar trends were observed in two other independent experiments. (B) Dose-dependent effects of different MAbs on the probability of single-cell adhesion measured between Cna^(+)^ cells and Cn substrates. Each data set represents the means of results of two independent experiments. All data were normalized to 100% frequency prior addition of MAbs.

Figure 6A shows the distribution of the maximum adhesion forces measured by SCFS between a Cna^(+)^ cell and a Cn substrate and its variation upon addition of the 9G7 MAb at increasing concentrations. The 9G7 MAb binds a conformational epitope located in the central region of Cna (residues 151 to 318) (14). Addition of 0.1 µg ml^−1^ lowered the adhesion frequency from 89% to 69%. While the weak forces (~800 pN) were moderately affected, many of the high forces (~3.2 nN) were abolished. This indicates that 9G7 interferes with the formation of strong collagen hug bonds. Increasing the concentration further decreased the adhesion probability to ~10%, most strong forces having been inhibited. By comparison, the 16H9 control MAb that does not recognize the ligand-binding region (14) did not substantially alter the adhesion probability, thus demonstrating the specific inhibition of Cna-mediated adhesion by 9G7. The concentration-dependent plot of 9G7 (Fig. 6B) documents an exponential decrease of adhesion probability, with the concentration required to inhibit 50% of maximum binding (IC_50_) being ~1 µg ml^−1^. While two other MAbs, 1H1 and 3D3, exhibited similar dose-dependent effects and IC_50_ values, 7C2 and 11H11 displayed a sharper drop in adhesion probability and lower IC_50_ values of around 0.2 µg ml^−1^. So our SCFS assay identified two inhibiting MAbs with enhanced antiadhesion activity. These MAbs may interact with Cna residues that are in direct contact with collagen and act as competitive inhibitors, while the others would bind outside the ligand binding site and interfere sterically with the Cna-Cn interaction. In future antiadhesion therapy, 7C2 and 11H11 could represent the most efficient MAbs for the prevention or treatment of staphylococcal infections.

## DISCUSSION

Cna is an MSCRAMM that can enhance the virulence of *S. aureus* and represents a potential target for antibacterial therapy. Despite the crucial role of Cna-mediated adhesion in pathogenesis, the underlying mechanism is not completely understood. Two unsolved questions are the following: what are the binding strength and elasticity of Cna in living bacterial cells, and what is the functional role of the B domain, if any? Through the use of advanced single-cell and single-molecule techniques, we have shown that Cna and Cn form very strong bonds *in vivo*, reflecting the collagen hug binding mechanism. We discovered that the B region exhibits a previously unidentified mechanical function, not yet described for any staphylococcal adhesin, that is required to activate strong ligand binding by the A region.

The mechanical strength and localization of single Cna adhesins were quantified in living bacteria. Two distinct types of Cna-Cn interactions were observed, i.e., weak bonds of ~250 pN attributable to hydrophobic interactions between Cn and the shallow trench of Cna and strong bonds of ~1,200 pN, reflecting fully secured collagen hug complexes. Recombinant fragments of the A region—comprising either the N1N2 subdomains or the N1N2N3 subdomains—form only weak bonds, thus supporting strongly the notion that the B region is required to activate the adhesive function of the protein. Cna is not randomly distributed over the bacterial surface but forms nanoscale domains. Interestingly, strong Cna-Cn bonds generally form small clusters, suggesting that collagen hug binding could be enhanced by cooperative interactions of multiple adhesins. We expect that, in analogy with fungal adhesins (35), densely packed adhesins within nanodomains generate stronger interactions, like those in multivalent systems, thus strengthening the attachment of whole cells to Cn substrates.

A remarkable observation of this study was that Cna exhibits novel and unanticipated mechanical properties. Under conditions involving force, the B region behaves as a stiff nanospring capable of withstanding high forces with minimal elongation. This finding favors a model in which the B region has an important mechanical function *in vivo*: the spring-like properties of the B repeats provide a means to project the protein binding sites away from the crowded molecular environment of the bacterial cell surface and to maintain cell adhesion under conditions of high mechanical stress. Depending on the cell environment, the spring may stretch and contract from the bacterial cell wall, thus modulating the adhesive function of Cna. We speculate that the CnaB-like folds of other MSCRAMMs may have evolved similar unique mechanical properties to fulfill their adhesive function.

Our finding that the B region is a stiff spring rationalizes earlier speculations about the putative function of the B repeats. Molecular modeling revealed that the B repeats pack in a zig-zag fashion, suggesting that they could effectively provide the needed orientation and stability to present the A region away from the bacterial cell surface (9). For the *S. aureus* SdrD protein, which also contains the CnaB-like fold (36), it has been proposed that the B repeats could function as a spacer and a spring, helping to expose the adhesin (36). Our observations differ from the conclusions of an earlier study on purified proteins, suggesting that the structure, function, and folding of the ligand-binding A region are not affected by the presence of the B region and thus that the latter does not influence collagen binding (7). So our work shows that the functional activity of recombinant domains *in vitro* may differ from that *in vivo*, highlighting the need to study functional adhesins directly on cell surfaces and thus in their biologically relevant conformation and environment.

What is the molecular origin of this unusual mechanical behavior? In light of earlier studies on the CnaB fold, including single-molecule stretching experiments, we postulate that the remarkable mechanical strength of the protein originates from the presence of internal isopeptide bonds. In Gram-positive bacterial pili, intramolecular isopeptide bonds within Cna B folds provide remarkable mechanical strength to the proteins (27, 28). Stretching experiments revealed that the *S. pyogenes* Spy0128 pilin is an inextensible protein, even when pulled at forces of up to 800 pN. This mechanical resilience results from strategically located intramolecular isopeptide bonds (29). AFM demonstrated that pili from the probiotic *Lactobacillus rhamnosus* GG bacterium exhibit nanospring properties (37). The pilus spring constant measured at high force (>500 pN) was 15 pN nm^−1^ and was thus similar to the Cna value. As there is evidence that isopeptide bonds may form in CnaB domains from *S. aureus* adhesins (30), we believe that the mechanical behavior of the B domain may result from the stabilization of the repeats by internal isopeptide bonds.

Finally, we have presented a valuable assay for assessing the antiadhesion activity of MAbs against staphylococcal adhesins. Unlike *in vitro* assays performed on purified molecules, AFM enables us to directly analyze the efficiency of a series of inhibitors without labeling or purification. We identified two inhibiting MAbs with enhanced antiadhesion activity which may interact with Cna residues that are in direct contact with collagen and act as competitive inhibitors. With the continuous growth of multiresistant strains, we expect that our new assay may find utility for the screening of efficient antiadhesion agents for therapy.

## MATERIALS AND METHODS

### Bacterial strains and cultures.

We used *Staphylococcus aureus* strain Phillips [Cna^(+)^ cells], originally isolated from a patient diagnosed with osteomyelitis, and its PH100 isogenic collagen adhesin-negative mutant [Cna^(−)^ cells] (2) in this study. Strains were grown in tryptic soy broth (TSB). For AFM experiments, cells from the stationary-growth phase (16 to 18 h) were harvested by 3 min of centrifugation at 2,500 × *g* and washed 2 times in phosphate-buffered saline (PBS) buffer. Stationary-phase cells express Cna very well.

### Collagen and recombinant proteins.

Type II collagen was purified from bovine nasal septum as described by Strawich and Nimni (38). CNA_31–344_ and CNA_31–531_ were produced as described elsewhere (5). Briefly, DNA encoding regions CNA_31–344_ and CNA_31–531_ was amplified by PCR using *S. aureus* FDA 574 genomic DNA as the template. Oligonucleotides were purchased from Integrated DNA Technologies (Leuven, Belgium). Restriction enzyme cleavage sites were incorporated at the 5′ ends of the primers to facilitate cloning into plasmid pQE30 (Qiagen, Chatsworth, CA). Restriction enzymes were purchased from New England Biolabs (Hertfordshire, United Kingdom). The integrity of cloned DNA was confirmed by sequencing (Primmbiotech, Milan, Italy).

### Monoclonal antibodies.

Monoclonal antibodies 9G7, 16H9, 11H11, 1H1, 3D3, and 7C2 raised against recombinant CNA_151-318_ were generated as described by Köhler and Milstein (39) with minor modifications and produced essentially as reported in reference 14. Briefly, BALB/c mice were injected intraperitoneally five times at 1-week intervals with 50 µg of the purified recombinant protein. The antigen was emulsified with an equal volume of complete Freund’s adjuvant for the first immunization, followed by three injections in incomplete adjuvant. The mice were bled, and the sera were tested for reactivity to the purified protein using ELISA and Western blotting. For the final immunization, the antigen was given in saline solution. Three days later, the lymphocytes were isolated from spleens and fused with Sp2/0-Ag14 mouse myeloma cells at a ratio of 5:1 using 50% (vol/vol) polyethylene glycol 4000. The suspended cells were first grown and selected in high-glucose Dulbecco’s modified Eagle’s medium (DMEM)/RPMI 1640 medium (Sigma) (1:1) containing 2% (wt/vol) hypoxanthine/aminopterin/thymidine (Sigma), 2% (wt/vol) glutamine, 2% (wt/vol) penicillin, and 2% (wt/vol) streptomycin. After 1 week, the hypoxanthine/aminopterin/thymidine medium was progressively replaced by culturing cloned hybridomas in a serum-free medium consisting of DMEM/RPMI 1640 supplemented with 1% (vol/vol) Nutridoma-S.R. (Roche Molecular Biochemicals) and antibiotics. Supernatants of the cell cultures were screened by ELISA on day 10, and hybridomas positive for the antibodies against B repeats were subcultured to a density of 1 cell per well by limiting dilution and further characterized by ELISA and Western blotting. The antibodies were purified using ammonium sulfate precipitation of the hybridoma supernatants, followed by affinity chromatography on a protein G-Sepharose column according to the recommendations of the manufacturer (GE Healthcare). Isotyping of the monoclonal antibodies produced was performed using a Mouse-Typer subisotyping kit (Bio-Rad). MAbs 16H9, 9G7, and 1H1 were IgG1-k, while MAbs 3D3, 7C2, and 11H11 were IgG2a-k.

### Collagen-coated tips and substrates.

To prepare Cn-coated tips and substrates for SMFS and SCFS experiments, gold-coated glass coverslips and cantilevers (OMCL-TR4; Olympus Ltd., Tokyo, Japan) were immersed overnight in an ethanol solution containing 1 mM 10% 16-mercaptododecahexanoic acid–90% 1-mercapto-1-undecanol (Sigma), rinsed with ethanol, and dried with N_2_. Tips and substrates were then immersed for 30 min in a solution containing 10 mg ml^−1^ *N*-hydroxysuccinimide (NHS) and 25 mg ml^−1^ 1-ethyl-3-(3-dimethylaminopropyl)-carbodiimide (EDC) (Sigma), rinsed 5 times with Ultrapure water (ELGA LabWater), incubated with 0.2 mg ml^−1^ of collagen type II for 1 h, rinsed further with PBS buffer, and then immediately used without dewetting.

### Adherence assays.

A microscopic adhesion assay was used to assess bacterial adhesion on Cn substrates. Substrates were incubated for 2 h in 200-µl bacterial suspensions adjusted in PBS to a concentration of 10^9^ cells ml^−1^. After 2 h, the substrates were gently rinsed by 3 consecutives washing in PBS and directly imaged using an inverted optical microscope (Zeiss Axio Observer Z1) equipped with a model C10600 Hamamatsu camera.

A crystal violet assay was also used. Microtiter wells were coated overnight at 4°C with 1 µg/well collagen type II–0.1 M sodium carbonate (pH 9.5). The plates were washed with 0.5% (vol/vol) PBS with Tween 20 (PBST). To block additional protein-binding sites, the wells were treated for 1 h at 22°C with 2% (vol/vol) bovine serum albumin (BSA)–phosphate-buffered saline (PBS). The wells were then incubated for 2 h at 37°C with 1 × 10^8^ *S. aureus* Phillips cells–PBS. After washing with PBS was performed, adhering cells were fixed with 2.5% formaldehyde for 30 min and stained with 1% crystal violet for 1 min. After washing, 100 µl of 10% acetic acid was added, and absorbance at 595 nm was recorded using an ELISA plate reader (BioRad).

### Single-molecule force spectroscopy.

SMFS measurements were performed at room temperature (20°C) in PBS buffer using a Nanoscope VIII Multimode AFM (Bruker Corporation, Santa Barbara, CA) and either oxide-sharpened microfabricated Si_3_Ni_4_ cantilevers with a nominal spring constant of ~0.01 N m^−1^ (MSCT) (Microlevers; Bruker Corporation) or gold-coated cantilevers with a nominal spring constant of ~0.02 N m^−1^ (OMCL-TR4; Olympus Ltd., Tokyo, Japan). The spring constants of the cantilevers were measured using the thermal noise method (Picoforce; Bruker).

For experiments performed with recombinant proteins, functionalized CNA_31–344_ and CNA_31–531_ tips were obtained using PEG-benzaldehyde linkers (23). Prior to functionalization, cantilevers were washed with chloroform and ethanol, placed in a UV-ozone cleaner for 30 min, immersed overnight in an ethanolamine solution (3.3 g ethanolamine–6 ml dimethyl sulfoxide [DMSO]), and then washed 3 times with DMSO and 2 times with ethanol and dried with N_2_. The ethanolamine-coated cantilevers were immersed for 2 h in a solution prepared by mixing 1 mg Acetal–PEG–NHS dissolved in 0.5 ml of chloroform with 10 µl triethylamine and then washed with chloroform and dried with N_2_. Cantilevers were further immersed for 5 min in a 1% citric acid solution, washed in Ultrapure water (ELGA LabWater), and then covered with a 200-µl droplet of PBS solution containing 200 µg/ml of the recombinant protein to which 2 µl of a 1 M NaCNBH_3_ solution was added. After 50 min, cantilevers were incubated with 5 µl of a 1 M ethanolamine solution in order to passivate unreacted aldehyde groups and then washed with and stored in buffer. In a control experiment, Cn-coated tips and CNA_31–344_-coated substrates were also prepared using NHS surface chemistry, as described above. Multiple force-distance curves were recorded between CNA tips (or Cn tips) and different spots of Cn substrates (or CNA substrates).

For live-cell experiments, bacteria were immobilized by mechanical trapping in porous polycarbonate membranes (Millipore, Billerica, MA) with a pore diameter of 0.8 µm. After filtration of a cell suspension was performed, the filter was gently rinsed with PBS, carefully cut into pieces (1 cm by 1 cm), and attached to a steel sample puck using a small piece of double-face adhesive tape, and the mounted sample was transferred into the AFM liquid cell while avoiding dewetting. Bare tips were first used to localize and image individual cells and then replaced by Cn tips prepared by the use of NHS chemistry as described above. Adhesion maps were obtained by recording 32-by-32 force-distance curves on areas of 500 by 500 nm, calculating the adhesion force for each force curve, and displaying adhesive events as red or green pixels. For blocking experiments, MAbs were added at 10 µg/ml.

### Single-cell force spectroscopy.

Bacterial cell probes were obtained as previously described (25, 26). Briefly, colloidal probes were obtained by attaching a single silica microsphere (Bangs Laboratories) (6.1-µm diameter) with a thin layer of UV-curable glue (NOA 63; Norland Edmund Optics) to triangle-shaped tipless cantilevers (NP-O10 Microlevers; Bruker Corporation) and using a Nanoscope VIII multimode AFM (Bruker Corporation, Santa Barbara, CA). The cantilever was then immersed for 1 h in a 10 mM Tris buffer–150 mM NaCl solution (pH 8.5) containing 4 mg ml^−1^ dopamine hydrochloride (Sigma) (99%). The probe was then rinsed in a Tris buffer–150 mM NaCl solution (pH 8.5) and used directly for cell probe preparation. The nominal spring constant of the colloidal probe cantilever as determined by the thermal noise method was ~0.06 N m^−1^.

For cell probe preparation, 50 µl of a suspension of ca. 1 × 10^6^ cells was transferred into a glass petri dish in which Cn-coated substrates were attached. Two milliliters of PBS was added to immerse bacteria and Cn substrates. The colloidal probe was brought into contact with an isolated bacterium. Single bacteria were attached on the center of the colloidal probes using a Bioscope Catalyst AFM (Bruker Corporation, Santa Barbara, CA) equipped with a Zeiss Z1 Axio Observer and a model C10600 Hamamatsu camera. The cell probe was then positioned over the Cn substrates without dewetting. Single-cell interaction forces with Cn substrates were measured at room temperature (20°C) by recording multiple force curves on five different spots. For blocking experiments, MAbs were added at increasing concentrations from 0.01 µg/ml to 10 µg/ml and the forces were measured after 15 min.

## SUPPLEMENTAL MATERIAL

Figure S1  Single-molecule force spectroscopy of CNA_31–531_. (A) Adhesion force histogram and (B) rupture length histogram with representative retraction force profiles obtained by recording force-distance curves in PBS between CNA_31–531_ tips and Cn substrates. CNA fragments were immobilized on the tips using a PEG-benzaldehyde linker. Data were pooled from independent experiments performed using 8 different tips and substrates. Download Figure S1, PDF file, 0.4 MB
