# Supplementary material for: Mechanical Strength and Inhibition of the Staphylococcus aureus Collagen-Binding Protein Cna
Source: mBio. 2016 Oct 25;7(5):e01529-16. doi: 10.1128/mBio.01529-16 (PMC5080380; doi:10.1128/mBio.01529-16)
Supplement: Figure S1 — Single-molecule force spectroscopy of CNA31–531. (A) Adhesion force histogram and (B) rupture length histogram with representative retraction force profiles obtained by recording force-distance curves in PBS between CNA31–531 tips and Cn substrates. CNA fragments were immobilized on the tips using a PEG-benzaldehyde linker. Data were pooled from independent experiments performed using 8 different tips and substrates. Download [file mbo005163050sf1.pdf]

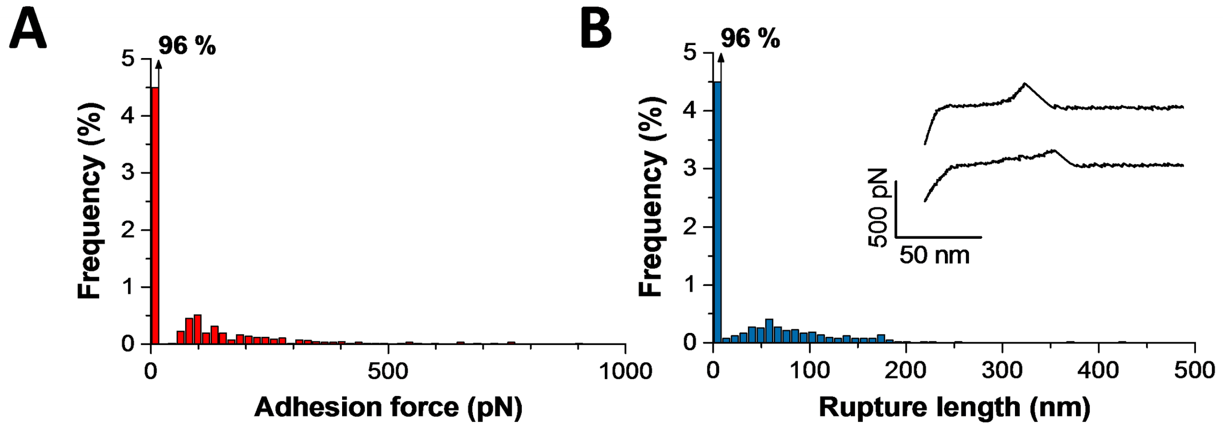

FIG S1. Single-molecule force spectroscopy of CNA$_{31\text{-}531}$. (A) Adhesion force histogram and (B) rupture length histogram with representative retraction force profiles obtained by recording force-distance curves in PBS between CNA$_{31\text{-}531}$-tips and Cn-substrates. CNA fragments were immobilized on the tips using a PEG-benzaldehyde linker. Data were pooled from independents experiments using 8 different tips and substrates.
